# Supplementary material for: Nanosecond-time-scale delayed fluorescence molecule for deep-blue OLEDs with small efficiency rolloff
Source: Nat Commun. 2020 Apr 14;11:1765. doi: 10.1038/s41467-020-15558-5 (PMC7156453; doi:10.1038/s41467-020-15558-5)
Supplement: Supplementary file 1 — Supplementary Information [file 41467_2020_15558_MOESM1_ESM.docx]

**Supplementary Information**

**Nanosecond-Time-Scale Delayed Fluorescence Molecule for Deep-Blue OLEDs with Small Efficiency Rolloff**

Jong Uk Kim,^a,b^ In Seob Park,^a^ Chin-Yiu Chan,^a^ Masaki Tanaka,^a^ Youichi Tsuchiya,^a^ Hajime Nakanotani,^a,b,c^ and Chihaya Adachi,*^a,b,c^

*^a^Center for Organic Photonics and Electronics Research (OPERA) and Department of Applied Chemistry, Kyushu University, 744 Motooka, Nishi-ku, Fukuoka 819-0395, Japan*

*^b^JST, ERATO, Adachi Molecular Exciton Engineering Project, Kyushu University, 744 Motooka, Nishi-ku, Fukuoka 819-0395, Japan*

*^c^International Institute for Carbon Neutral Energy Research (WPI-I^2^CNER), Kyushu University, 744 Motooka, Nishi-ku, Fukuoka 819-0395, Japan*

**Corresponding author*

*E-mail address: adachi@cstf.kyushu-u.ac.jp*

**Supplementary Figures**


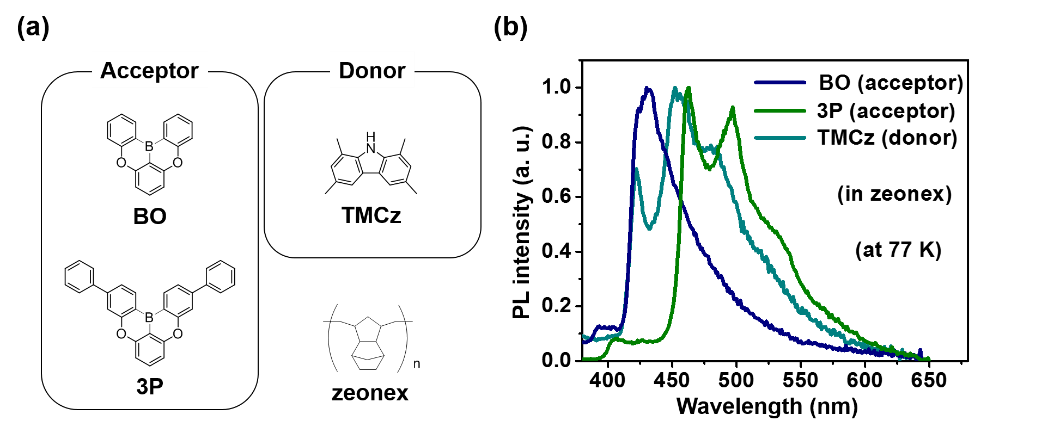


**Supplementary Figure 1 | PL of composite molecules.** (a) Molecular structures of **BO**, **3P** (acceptor), and **TMCz** (donor) (b) Phosphorescence (77 K) spectra of **BO**, **3P** (acceptor), and **TMCz** (donor).


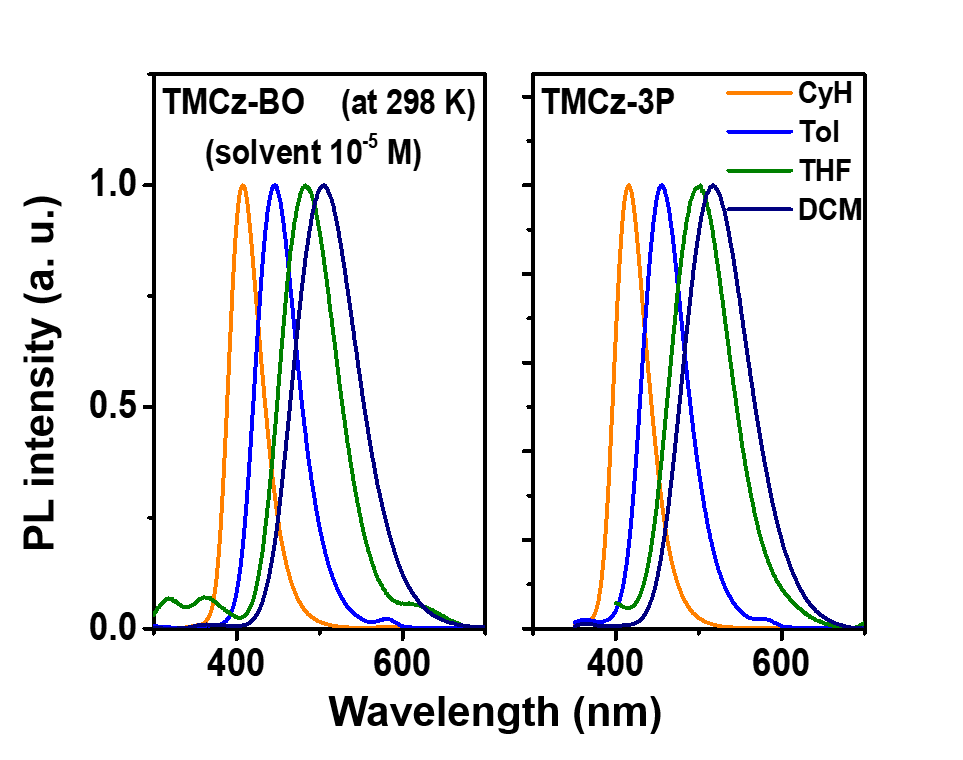


**Supplementary Figure 2 |** **Solvent dependence of PL.** Fluorescence spectra with bathochromic shifts of **TMCz-BO** and **TMCz-3P** in cyclohexane (CyH), toluene (Tol), tetrahydrofuran (THF), and dichloromethane (DCM) solutions.


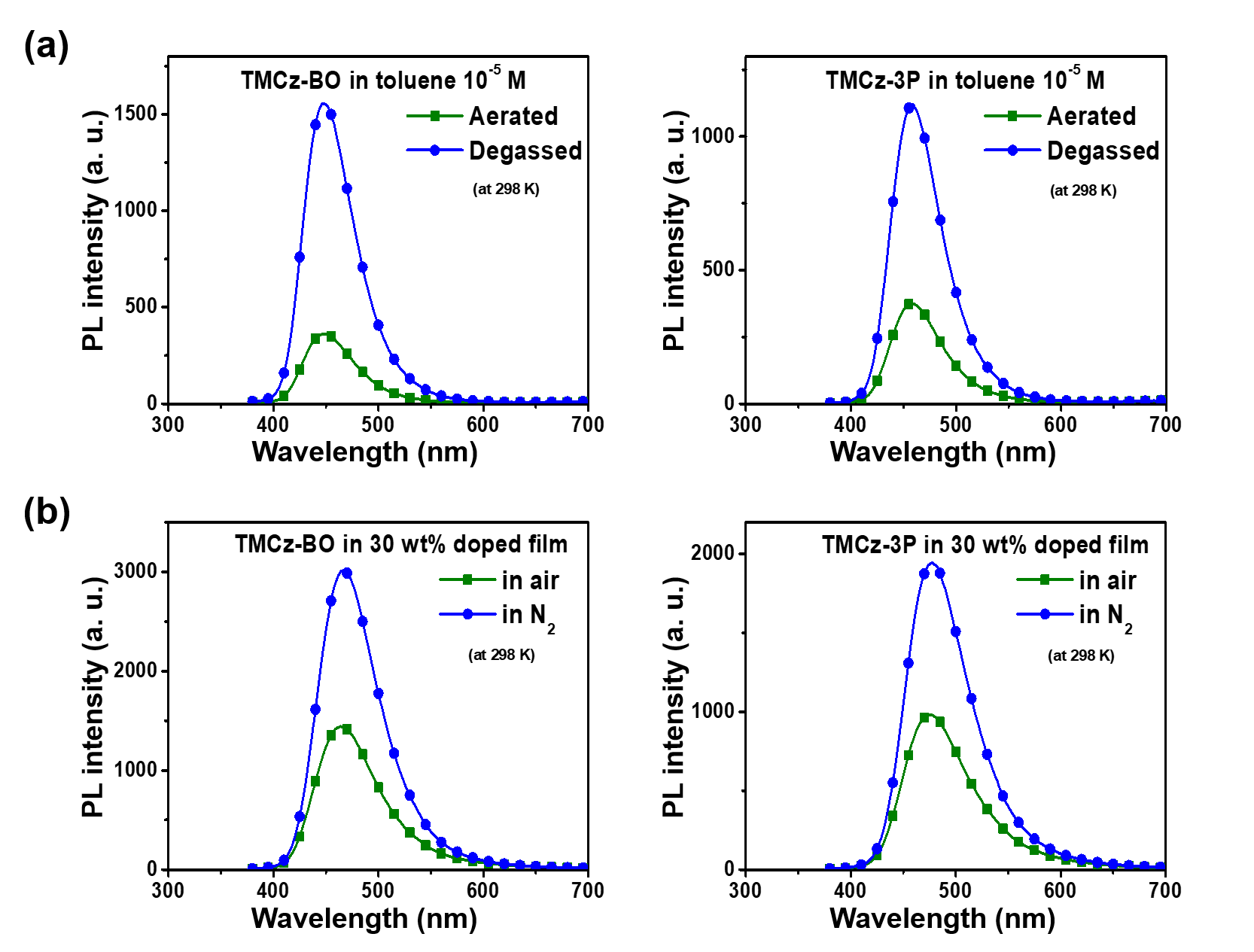


**Supplementary Figure 3 |** **Oxygen dependence of PL spectra for** **TMCz-BO** and **TMCz-3P.** (a) toluene solution (10^−5^ M) and (b) 30 wt% doped films in a PPF host matrix.


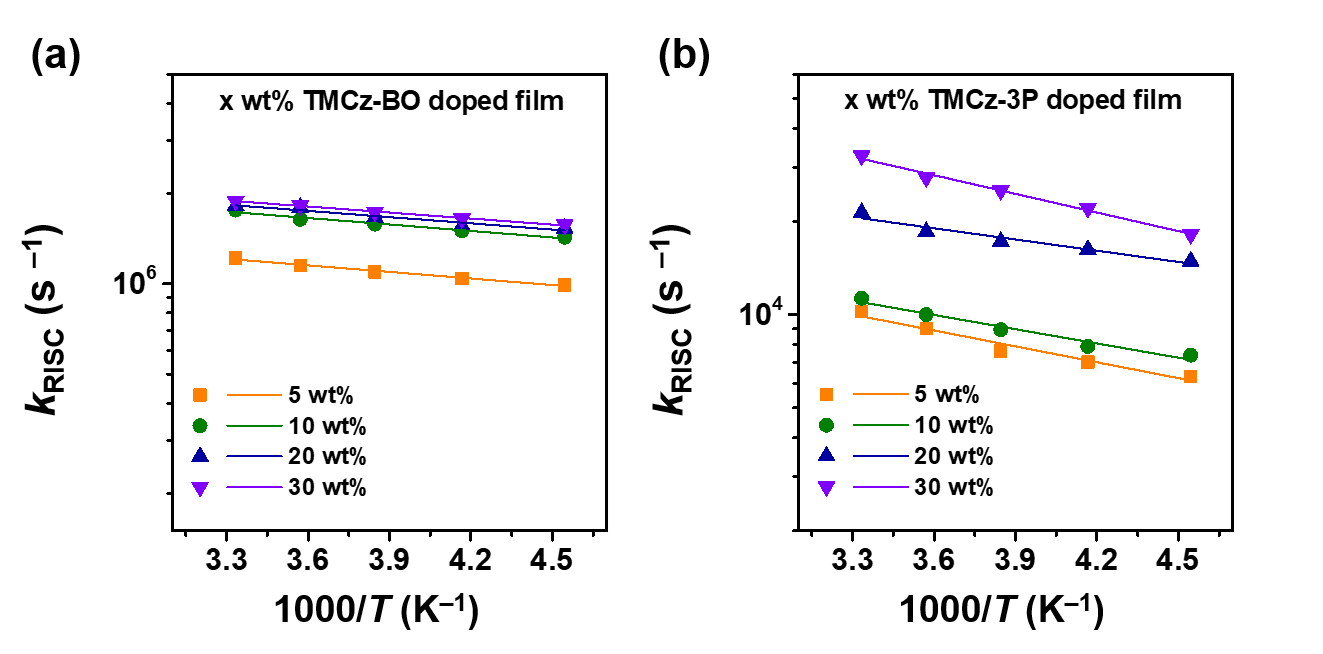


**Supplementary Figure 4 |** **Arrhenius plots of the rate constants of RISC (*k*_RISC_).** *k*_RISC_ was obtained with x=5(■), 10(●), 20 (▲), and 30 (▼) wt% doped films of (a) **TMCz-BO** and (b) **TMCz-3P**, where the solid lines denote the least-squares fittings.


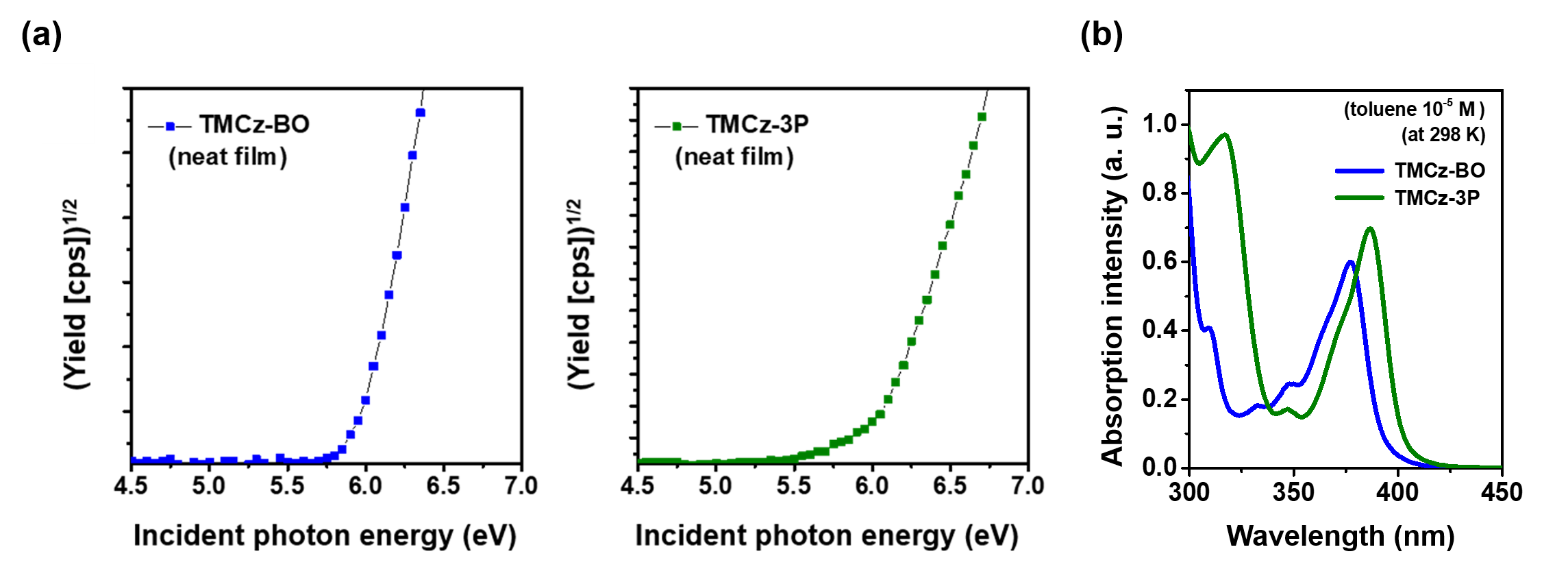


**Supplementary Figure 5 |** **HOMO and LUMO estimation.** (a) Photoelectron yield spectra (in films) and (b) absorption spectra (in Tol solution 10^−5^ M) of **TMCz-BO** and **TMCz-3P**.


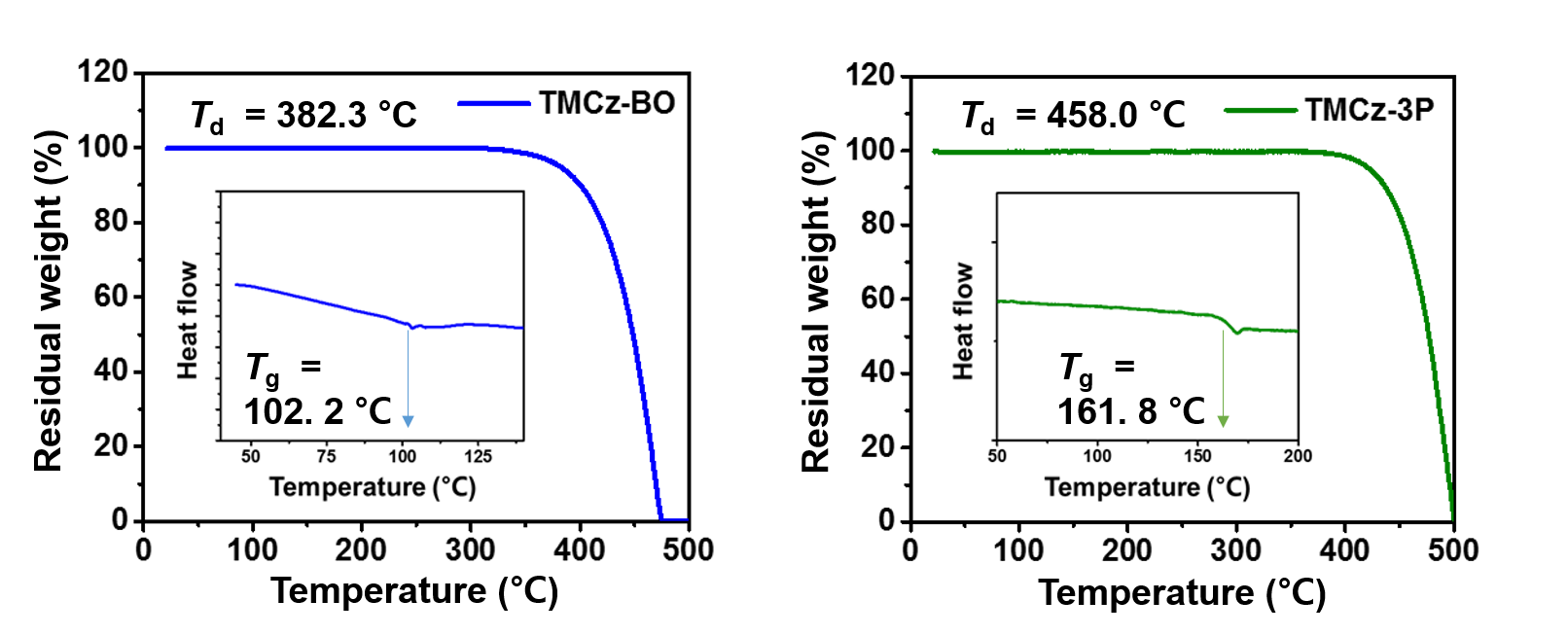


(b)

(a)

**Supplementary Figure 6 | Thermal properties of thin films.** TGA and DSC (inset) curves for (a) **TMCz-BO** and (b) **TMCz-3P** recorded at a heating rate of 5 °C min^−1^ under N_2_. *T*_d_ is the decomposition temperature, corresponding to 5% weight loss upon heating and *T*_g_ is the glass-transition temperature.


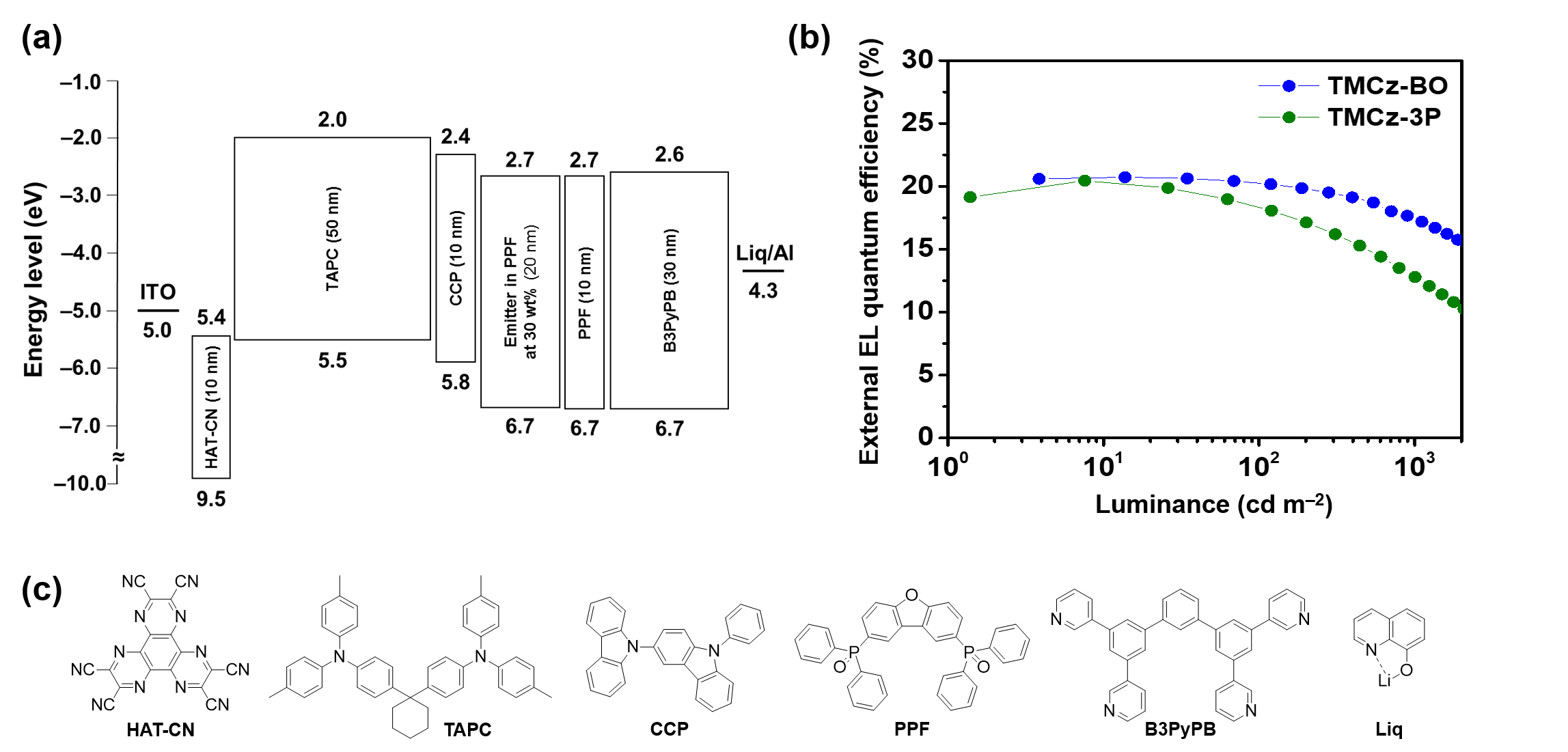


**Supplementary Figure 7 | OLED characteristics.** (a) Energy level diagram and (b) external EL quantum efficiency (*η*_ext_) versus *L* plots of devices based on **TMCz-BO** and **TMCz-3P**. (c) Molecular structures of materials used for TADF-OLEDs.

**Supplementary Tables**

**Supplementary Table 1 | Summary of TDDFT calculations (PBE0/6-31G(d)).**

| Compound | HOMO [eV] | LUMO [eV] | *E*_g_ [eV] | *E*_S_ / *E*_T_ [eV] | Δ*E*_ST_ [eV] | *f* @S_0_ | *μ* [D] | *λ*_S_ [eV] |
| --- | --- | --- | --- | --- | --- | --- | --- | --- |
| **TMCz-BO** | −5.29 | −1.83 | 3.46 | 2.56 / 2.55 | 0.01 | 0.0057 | 3.39 | 0.15 |
| **TMCz-3P** | −5.28 | −1.93 | 3.35 | 2.47 / 2.46 | 0.01 | 0.0013 | 3.83 | 0.16 |

**Supplementary Table 2 | Activation energy of TMCz-BO and TMCz-3P.**

| TADF emitter | x wt% **TMCz-BO** doped film in PPF | | | | x wt% **TMCz-3P** doped film in PPF | | | |
| --- | --- | --- | --- | --- | --- | --- | --- | --- |
|  | 5 wt% | 10 wt% | 20 wt% | 30 wt% | 5 wt% | 10 wt% | 20 wt% | 30 wt% |
| *E*_a,RISC_^a)^ [meV] | 14.5 | 14.2 | 13.7 | 13.4 | 34.0 | 30.4 | 24.0 | 39.8 |
| y-intercept [s^−1^] | 2.1×10^−6^ | 3.0×10^−6^ | 3.1×10^−6^ | 3.3×10^−6^ | 3.6×10^−4^ | 3.6×10^−4^ | 5.4×10^−4^ | 15×10^−4^ |

1. Activation energies of the rate constants of RISC (*k*_RISC_) obtained with the x wt% doped films of **TMCz-BO** and **TMCz-3P**.

**Supplementary Table 3 | Photophysical data of TMCz-BO and TMCz-3P.** Rate constants and quantum efficiencies of **TMCz-BO** and **TMCz-3P** in 30 wt%-doped films^a)^

| Compound | *k*_r_  [s^-1^] | *k*_ISC_  [s^-1^] | *k*_RSC_  [s^-1^] | *Φ*_p_  [%] | *Φ*_d_  [%] | *Φ*_ISC_  [%] | *Φ*_RISC_  [%] |
| --- | --- | --- | --- | --- | --- | --- | --- |
| **TMCz-BO** | 1.7×10^7^ | 0.9×10^7^ | 1.9×10^6^ | 66 | 32 | 34 | 96 |
| **TMCz-3P** | 2.3×10^7^ | 1.2×10^7^ | 3.3×10^4^ | 65 | 11 | 35 | 41 |

^a)^Abbreviations: *k*_r_, radiative rate constant (S_1_→S_0_); *k*_ISC_, intersystem-crossing (ISC) rate constant (S_1_→T_1_); *k*_RISC_, reverse ISC rate constant (T_1_→S_1_); *Φ*_p_, quantum efficiency for prompt fluorescence component; *Φ*_d_, quantum efficiency for delayed fluorescence component; *Φ*_ISC_, ISC quantum efficiency; *Φ*_RISC_, RISC quantum efficiency.

**Supplementary Table 4 | EL performance of deep-blue (CIE*y* ≤ 0.20) TADF-based OLEDs.**

| TADF emitter | *λ*_EL_  [nm] | CIE (*x*, *y*) | *η*_ext_ (%) | | | | *η*_ext_ decrease ratio (%) | | | Ref. |
| --- | --- | --- | --- | --- | --- | --- | --- | --- | --- | --- |
|  |  |  | max | @100  cd m^−2^ | @500  cd m^−2^ | @1000  cd m^−2^ | @100  cd m^−2^ | @500  cd m^−2^ | @1000 cd m^−2^ |  |
| **TMCz-BO** | 471 | (0.14, 0.18) | 20.7 | 20.2 | 18.7 | 17.4 | 2.4 | 9.7 | 15.9 | ♣ |
| ***v*-DABNA** | 469 | (0.12, 0.11) | 34.4 | 32.8 |  | 26.0 | 4.7 |  | 24.4 | Ref.1 |
| **TDBA-Ac** | 448 | (0.14, 0.15) | 25.7 | >22 |  | 18.9 |  |  | 26.4 | Ref.2 |
| **TDBA-DI** |  | (0.14, 0.15) | 32.2 | >18 |  | 26.8 |  |  | 17.0 |  |
| **DPAc-DtCzBN** | 456 | (0.16, 0.15) | 23.1 | 18.3 |  |  | 20.8 |  |  | Ref.3 |
| **B2** | 460 | (0.13, 0.11) | 18.3 | 12.6 |  |  | 31.1 |  |  | Ref.4 |
| **PX-SBA** | 448 | (0.16, 0.13) | 20.8 | 14.2 |  | 7.0 | 31.7 |  | 66.3 | Ref.5 |
| **TTAZ** | 464 | (0.15, 0.16) | 23.7 | 14.5 | 4.7 |  | 38.8 | 80.4 |  | Ref.6 |
| **TXAZ** | 456 | (0.15, 0,13) | 16.0 | 8.5 |  |  | 47.0 |  |  |  |
| **2** | 462 | (0.16, 0.20) | 12.2 | 8.2 |  |  | 32.8 |  |  | Ref.7 |
| **4** | 460 | (0.16, 0.19) | 14.3 | 8.4 |  |  | 41.3 |  |  |  |
| **5** | 458 | (0.15, 0.15) | 11.4 | 5.4 |  |  | 52.6 |  |  |  |
| **DCzTrz** |  | (0.15, 0.16) | 17.8 | >16 | 14.9 |  |  | 16.3 |  | Ref.8 |
| **DMOC-DPS** | 460 | (0.16, 0.16) | 14.5 | 1.5 |  |  | 89.7 |  |  | Ref.9 |
| **DCzIPN** | 462 | (0.17, 0.19) | 16.4 | >15 |  |  |  |  |  | Ref.10 |
| **3CzFCN** |  | (0.16, 0.19) | 17.8 | 10.9 |  |  | 38.8 |  |  | Ref.11 |
| **TPXZPO** | 464 | (0.17, 0.20) | 15.3 |  |  |  | 14.0 |  | 41.0 | Ref.12 |
| **DTPDDA** | 468 | (0.15, 0.20) | 22.3 |  |  | 10.6 |  |  | 52.5 | Ref.13 |
| **DABNA-2** | 468 | (0.12, 0.13) | 20.2 | 13.4 |  |  | 33.7 |  |  | Ref.14 |
| **DMAC-DPS** | 470 | (0.16, 0.20) | 19.5 |  |  | 16.0 |  |  | 17.9 | Ref.15 |
| **2** |  |  | 19.0 |  |  |  |  |  |  | Ref.16 |
| **3** |  | (0.14, 0.16) | 20.1 |  |  |  |  |  |  |  |
| **4** |  | (0.14, 0.16) | 13.3 |  |  |  |  |  |  |  |
| **3DPyM-pDTC** | 464 | (0.14, 0.18) | 31.9 | 26.1 | 20.1 |  | 18.2 | 37.0 |  | Ref.17 |
| **Cz-TRZ3** |  | (0.15, 0.10) | 19.2 |  |  |  |  |  |  | Ref.18 |
| **Cz-TRZ4** |  | (0.15, 0.10) | 18.3 |  |  | 8.0 |  |  | 56.3 |  |
| **4CzBN** | 458 | (0.17, 0.20) | 10.6 |  | 5.4 |  |  | 49.1 |  | Ref.19 |
| **34TCzTTrz** | 463 | (0.16, 0.20) | 10.3 | >4 |  |  |  |  |  | Ref.20 |
| **10** | 466 |  | 15.1 | >7 |  |  |  |  |  | Ref.21 |
| **CzoB** | 466 | (0.14, 0.15) | 22.6 | 18.4 |  | 5.1 | 18.6 |  | 77.4 | Ref.22 |
| **ICzDAc** |  | (0.15, 0.16) | 19.5 |  |  | >10 |  |  |  | Ref.23 |
| **Ac-3MHPM** | 454 | (0.16, 0.15) | 17.8 | 10.4 |  |  | 41.6 |  |  | Ref.24 |
| **DCzBN3** | 428 | (0.16, 0.06) | 10.3 | 5.4 |  |  | 47.6 |  |  | Ref.25 |
| **MA-TA** |  | (0.16, 0.19) | 22.1 | 13.9 |  |  | 37.1 |  |  | Ref.26 |
| **3 (CzoB)** | 473 | (0.14, 0.20) | 24.1 | 19.6 | 11.4 |  | 18.7 |  |  | Ref.27 |
| **4 (CzMeoB)** | 470 | (0.14, 0.14) | 18.4 | 15.3 | 8.9 |  | 16.8 |  |  |  |
| **CNBPCz** | 456 | (0.14, 0.12) | 14 | >1 |  |  |  |  |  | Ref.28 |
| **CNICCz** | 449 | (0.15, 0.08) | 12.4 | 6.4 |  |  | 48.4 |  |  | Ref.29 |
| **CNICtCz** | 456 | (0.14, 0.13) | 16 | 10.7 |  |  | 33.1 |  |  |  |
| **OBA-O (A2)** | 446 | (0.17, 0.17) | 17.8 | 15.5 |  | 8.5 | 12.9 |  | 52.2 | Ref.30 |
| **III** |  | (0.15, 0.18) | 22.6 | 22.5 |  | 19.9 | 0.4 |  | 11.9 | Ref.31 |

**Supplementary methods**

The synthetic routes for intermediates **1**–**4** are outlined in **Supplementary Figures 8-11**. The detailed synthetic procedures and characterization data for intermediates **1**–**4** are described below.

**
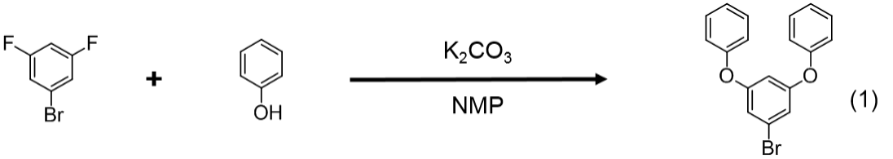
**

**Supplementary Figure 8 | Synthesis of compound 1.**

**Synthesis of 5-bromo-1,3-diphenoxybenzene (1)**: NMP (150 mL) was added to a mixture of 1-bromo-3,5-difluorobenzene (20.0 g, 0.10 mol), phenol (24.5 g, 0.26 mol), and K_2_CO_3_ (35.9 g, 0.26 mol) at room temperature under a nitrogen atmosphere. The reaction mixture was stirred at 170 ºC for 12 h and then cooled to room temperature. After the addition of toluene and water for extraction, the organic layer was washed with brine, dried over anhydrous MgSO_4_, and then condensed in vacuo. The crude product was purified using silica gel column chromatography (eluent; hexane/dichloromethane = 5/1) to afford compound **1** (28.3 g, 80% yield) as a colourless liquid. ^1^H NMR (500 MHz, CDCl_3_, *δ*): 7.29 (td, *J* = 7.1, 1.7 Hz, 4H), 7.08 (tt, *J* = 7.4, 2.1 Hz, 2H), 6.96 (dd, *J* = 8.7, 2.1 Hz, 4H), 6.74 (d, *J* = 2.2 Hz, 2H), 6.52 (t, *J* = 2.2 Hz, 1H); MS (ASAP) *m/z*: [*M*+1]^+^ calcd 341.20; found, 342.00.

**
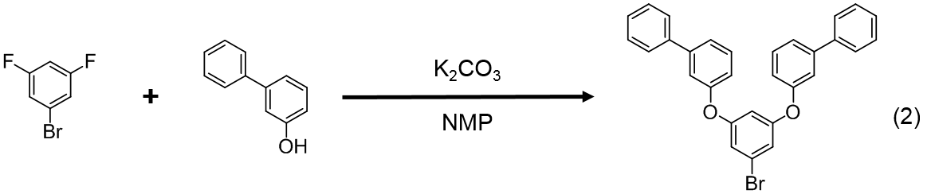
**

**Supplementary Figure 9 | Synthesis of compound 2.**

**Synthesis of 3,3''-((5-bromo-1,3-phenylene)bis(oxy))di-1,1'-biphenyl (2)**: Compound **2** was synthesized using the same procedure described above for compound **1**. 3-phenylphenol (44.0 g, 0.26 mol) was used instead of phenol, giving compound **2** (40.9 g, 80% yield) as a colourless liquid. ^1^H NMR (500 MHz, CDCl_3_, *δ*): 7.49 (dd, *J* = 8.45, 1.2 Hz, 4H), 7.36 (t, *J* = 7.5 Hz, 6H), 7.33-7.27 (m, 4H), 7.20 (t, *J* = 2.0 Hz, 2H), 6.94 (m, 2H), 6.81 (d, *J* = 2.2 Hz, 2H), 6.60 (t, *J* = 2.2 Hz, 1H); MS (ASAP) *m/z*: [*M*+1]^+^ calcd 493.39; found, 495.05.


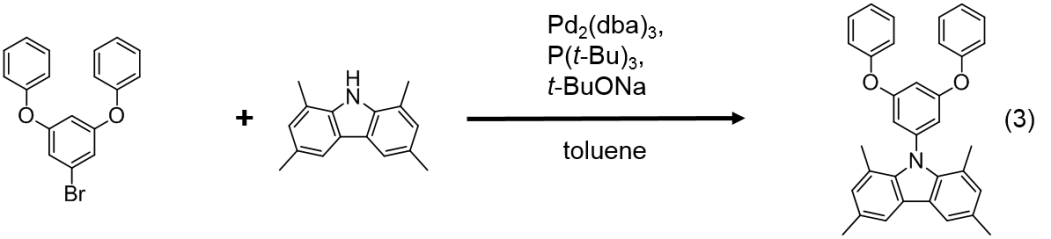


**Supplementary Figure 10 | Synthesis of compound 3.**

**Synthesis of 9-(3,5-diphenoxyphenyl)-1,3,6,8-tetramethyl-9*H*-carbazole (3)**: To a solution of **1** (20.0 g, 58.6 mmol) in toluene (100 mL), 1,3,6,8-tetramethyl-9*H*-carbazole (14.4 g, 64.5 mmol), Pd_2_(dba)_3_ (2.68 g, 2.93 mmol), P(*t*-Bu)_3_ (1.19 g, 5.86 mmol), and *t*-BuONa (12.4 g, 12.9 mmol) were added. After stirring at 110 ºC for 6 h, water was added to the reaction mixture at room temperature. The combined organic layer was extracted with toluene and then dried over anhydrous MgSO_4_. After filtration and evaporation, the crude product was purified by column chromatography on silica gel (eluent; hexane/dichloromethane = 5/1) to afford compound **3** (22.7 g, 80% yield) as a white solid. ^1^H NMR (500 MHz, CDCl_3_, *δ*): 7.70 (d, *J* = 8.1 Hz, 2H), 7.35 (td, *J* = 7.0, 1.7 Hz, 4H), 7.13 (tt, *J* = 7.4, 2.1 Hz, 2H), 7.07 (dt, *J* = 7.8, 1.5 Hz, 4H), 6.94 (s, 2H), 6.90 (t, *J* = 4.5 Hz, 1H), 6.81 (d, *J* = 2.3 Hz, 2H) 2.47 (s, 6H), 2.08 (s, 6H); MS (ASAP) *m/z*: [*M*+1]^+^ calcd 483.60; found, 485.17.


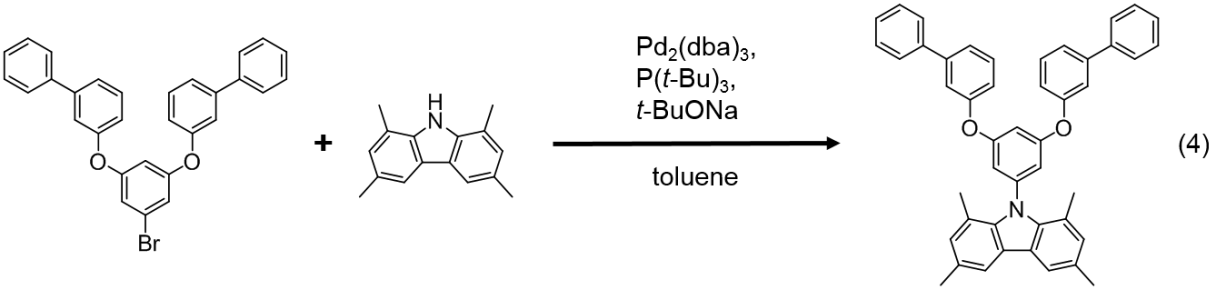


**Supplementary Figure 11 | Synthesis of compound 4.**

**Synthesis of 9-(3,5-bis([1,1'-biphenyl]-3-yloxy)phenyl)-1,3,6,8-tetramethyl-9*H*-carbazole (4)**: Compound **4** was synthesized using the same procedure described above for **TMCz-BO**, except that **2** (20.0 g, 40.5 mmol), 1,3,6,8-tetramethyl-9*H*-carbazole (9.96 g, 44.5 mmol), Pd_2_(dba)_3_ (1.86 g, 2.03 mmol), P(*t*-Bu)_3_ (0.82 g, 4.05 mmol), and *t*-BuONa (8.57 g, 89.2 mmol) were used as the reactants, giving compound **4** (20.5 g, 80% yield) as a light-yellow solid. ^1^H NMR (500 MHz, CDCl_3_, *δ*): 7.70 (s, 2H), 7.53 (dd, *J* = 8.5, 1.2 Hz, 4H), 7.46-7.41 (m, 5H), 7.39-7.34 (m, 5H), 7.29 (t, *J* = 4.0 Hz, 2H), 7.06-7.03 (m, 2H), 6.98 (t, *J* = 4.5 Hz, 1H), 6.93(s, 2H), 6.91(d, *J* = 2.3 Hz, 2H), 2.47(s, 6H), 2.10(s, 6H); MS (ASAP) *m/z*: [*M*]^+^ calcd 635.79; found, 635.27.


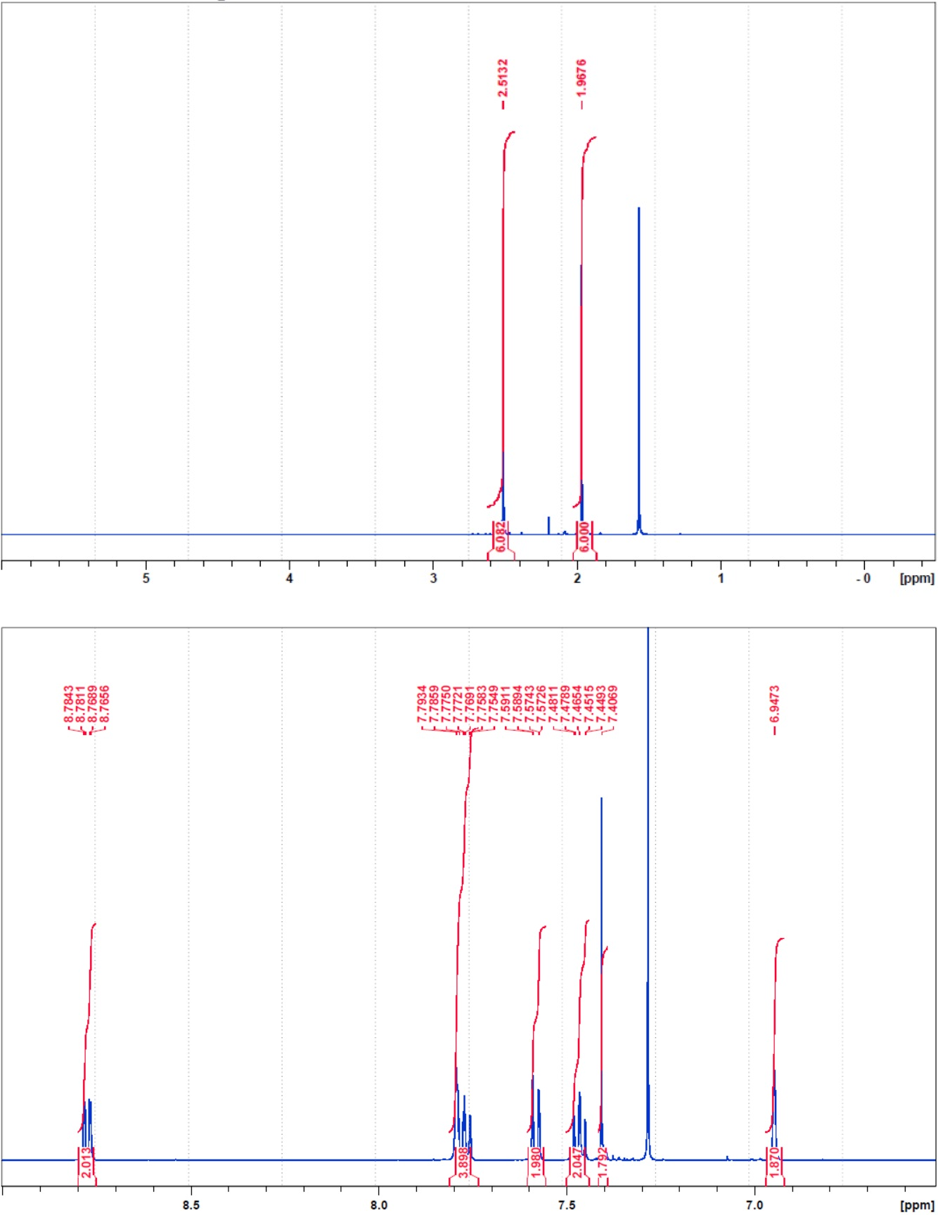


**Supplementary Figure 12 | ^1^H NMR spectrum of TMCz-BO in CDCl_3_.**


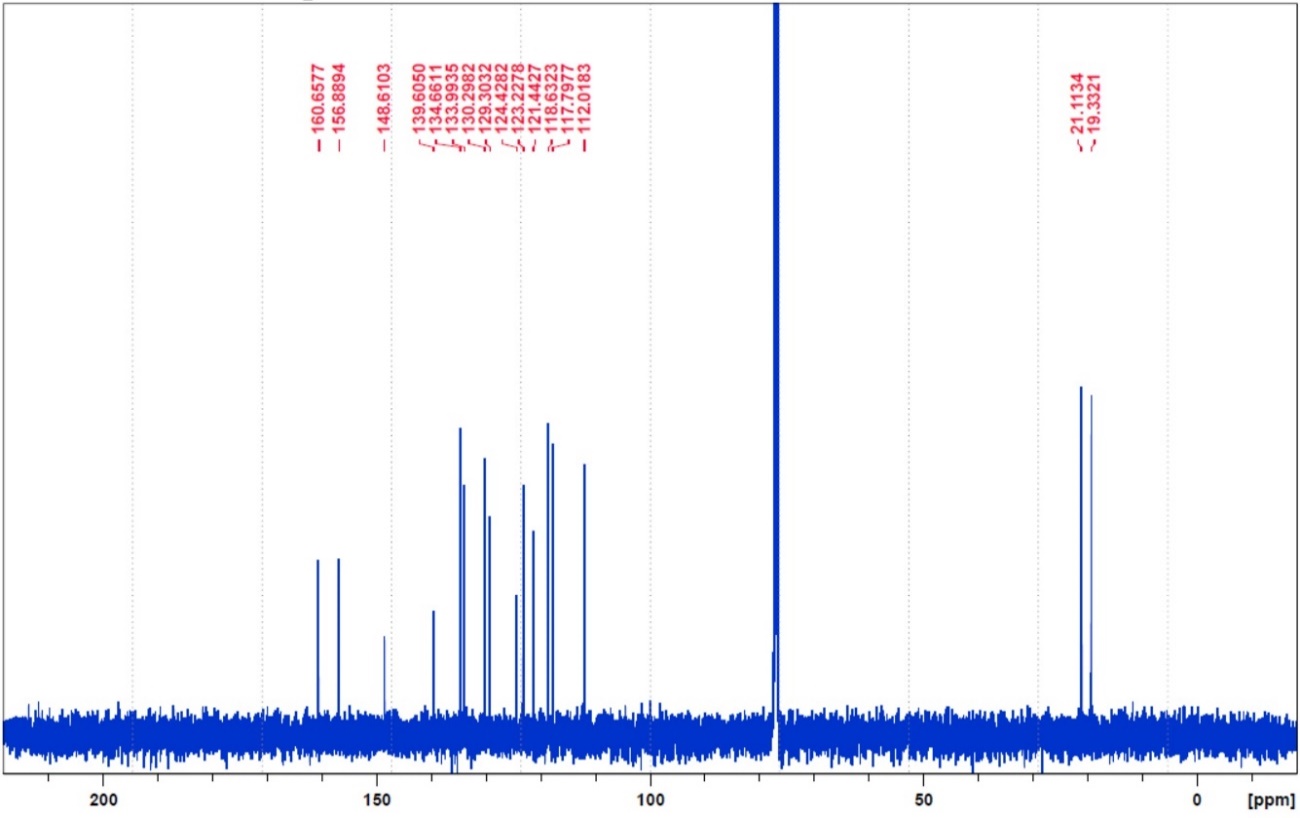


**Supplementary Figure 13 | ^13^C NMR spectrum of TMCz-BO in CDCl_3_.**


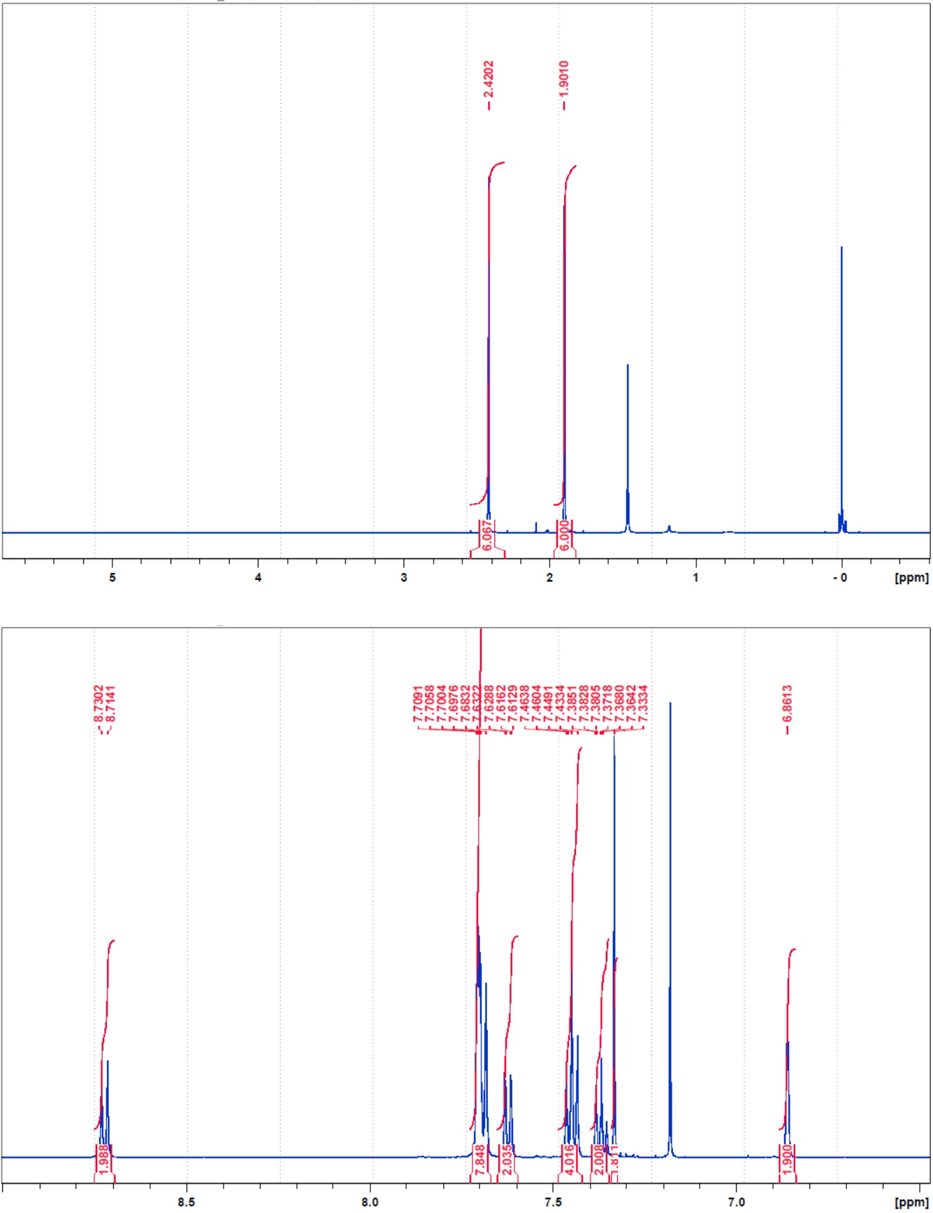


**Supplementary Figure 14 | ^1^H NMR spectrum of TMCz-3P in CDCl_3_.**


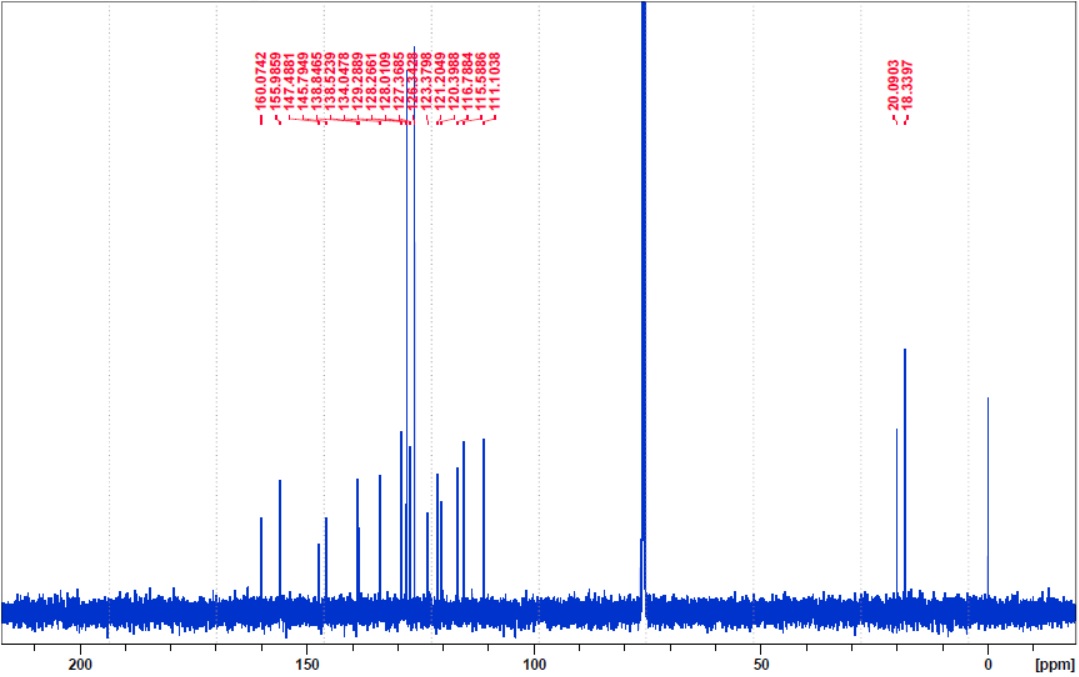


**Supplementary Figure 15 | ^13^C NMR spectrum of TMCz-3P in CDCl_3_.**

**Supplementary References**

1. Kondo, Y. et al*.* Narrowband deep-blue organic light-emitting diode featuring an organoboron-based emitter. *Nat. Photonics* **13**, 678–682 (2019).
2. Ahn, D. H. et al*.* Highly efficient blue thermally activated delayed fluorescence emitters based on symmetrical and rigid oxygen-bridged boron acceptors. *Nat. Photonics* **13**, 540–546 (2019).
3. Cheng, Z. et al*.* Achieving efficient blue delayed electrofluorescence by shielding acceptors with carbazole units. *ACS Appl. Mater. Interfaces* **11**, 28096–28105 (2019).
4. Matsui, K. et al*.* One-shot multiple borylation toward BN-doped nanographenes. *J. Am. Chem. Soc.* **140**, 1195–1198 (2018).
5. Liu, M. et al*.* Horizontally orientated sticklike emitters: enhancement of intrinsic out-coupling factor and electroluminescence performance. *Chem. Mater.* **29**, 8630–8636 (2017).
6. Woo, S. J., Kim, Y., Kwon, S. K., Kim, Y. H. & Kim, J. J. Phenazasiline/spiroacridine donor combined with methyl-substituted linkers for efficient deep blue thermally activated delayed fluorescence emitters. *ACS Appl. Mater. Interfaces* **11**, 7199–7207 (2019).
7. Park, I. S., Komiyama, H. & Yasuda, T. Pyrimidine-based twisted donor-acceptor delayed fluorescence molecules: a new universal platform for highly efficient blue electroluminescence. *Chem. Sci.* **8**, 953–960 (2017).
8. Kim, M., Jeon, S. K., Hwang, S. H. & Lee, J. Y. Stable blue thermally activated delayed fluorescent organic light-emitting diodes with three times longer lifetime than phosphorescent organic light-emitting diodes. *Adv. Mater.* **27**, 2515–2520 (2015).
9. Wu, S. et al*.* High-efficiency deep-blue organic light-emitting diodes based on a thermally activated delayed fluorescence emitter. *J. Mater. Chem. C* **2**, 421–424 (2014).
10. Cho, Y. J., Yook, K. S. & Lee, J. Y. Cool and warm hybrid white organic light-emitting diode with blue delayed fluorescent emitter both as blue emitter and triplet host. *Sci. Rep.* **5**, 7859 (2015).
11. Cho, Y. J., Chin, B. D., Jeon, S. K. & Lee, J. Y. 20% External quantum efficiency in solution-processed blue thermally activated delayed fluorescent devices. *Adv. Funct. Mater.* **25**, 6786–6792 (2015).
12. Duan, C. et al. Multi-dipolar chromophores featuring phosphine oxide as joint acceptor: a new strategy toward high-efficiency blue thermally activated delayed fluorescence dyes. *Chem. Mater.* **28**, 5667–5679 (2016).
13. Sun, J. W. et al. Thermally activated delayed fluorescence from azasiline based intramolecular charge-transfer emitter (DTPDDA) and a highly efficient blue light emitting diode. *Chem. Mater.* **27**, 6675–6681 (2015).
14. Hatakeyama, T. et al*.* Ultrapure blue thermally activated delayed fluorescence molecules: efficient HOMO-LUMO separation by the multiple resonance effect. *Adv. Mater.* **28**, 2777–2781 (2016).
15. Zhang, Q. et al. Efficient blue organic light-emitting diodes employing thermally activated delayed fluorescence. *Nat. Photonics* **8**, 326–332 (2014).
16. Numata, M., Yasuda, T. & Adachi, C. High efficiency pure blue thermally activated delayed fluorescence molecules having 10*H*-phenoxaborin and acridan units. *Chem. Commun.* **51**, 9443–9446 (2015).
17. Rajamalli, P. et al*.* New molecular design concurrently providing superior pure blue, thermally activated delayed fluorescence and optical out-coupling efficiencies. *J. Am. Chem. Soc.* **139**, 10948–10951 (2017).
18. Cui, L. S. et al*.* Controlling singlet–triplet energy splitting for deep-blue thermally activated delayed fluorescence emitters. *Angew. Chem. Int. Ed.* **56**, 1571–1575 (2017).
19. Zhang, D., Cai, M., Zhang, Y., Zhang, D. & Duan, L. Sterically shielded blue thermally activated delayed fluorescence emitters with improved efficiency and stability. *Mater. Horizons* **3**, 145–151 (2016).
20. Kim, M. et al*.* Correlation of molecular structure with photophysical properties and device performances of thermally activated delayed fluorescent emitters. *J. Phys. Chem. C* **120**, 2485–2493 (2016).
21. Kitamoto, Y. et al*.* Light blue and green thermally activated delayed fluorescence from 10*H*-phenoxaborin-derivatives and their application to organic light-emitting diodes. *J. Mater. Chem. C* **3**, 9122–9130 (2015).
22. Lee, Y. H. et al*.* Rigidity-induced delayed fluorescence by ortho donor-appended triarylboron compounds: record-high efficiency in pure blue fluorescent organic light-emitting diodes. *ACS Appl. Mater. Interfaces* **9**, 24035–24042 (2017).
23. Seo, J. A., Im, Y., Han, S. H., Lee, C. W. & Lee, J. Y. Unconventional molecular design approach of high-Efficiency deep blue thermally activated delayed fluorescent emitters using indolocarbazole as an acceptor. *ACS Appl. Mater. Interfaces* **9**, 37864–37872 (2017).
24. Komatsu, R. et al*.* Manipulating the electronic excited state energies of pyrimidine-based thermally activated delayed fluorescence emitters to realize efficient deep-blue emission. *ACS Appl. Mater. Interfaces* **9**, 4742–4749 (2017).
25. Chan, C.-Y., Cui, L.-S., Kim, J. U., Nakanotani, H. & Adachi, C. Rational molecular design for deep-blue thermally activated delayed fluorescence emitters. *Adv. Funct. Mater.* **28** 1706023 (2018).
26. Wada, Y., Kubo, S. & Kaji, H. Adamantyl substitution strategy for realizing solution-processable thermally stable deep-blue thermally activated delayed fluorescence materials. *Adv. Mater.* **30**, 1705641 (2018).
27. Lee, Y. H. et al*.* High-efficiency sky blue to ultradeep blue thermally activated delayed fluorescent diodes based on ortho-carbazole-appended triarylboron emitters: above 32% external quantum efficiency in blue devices. *Adv. Opt. Mater.* **6**, 1800385 (2018).
28. Cho, Y. J., Jeon, S. K., Lee, S. S., Yu, E. & Lee, J. Y. donor interlocked molecular design for fluorescence-like narrow emission in deep blue thermally activated delayed fluorescent emitters. *Chem. Mater.* **28**, 5400–5405 (2016).
29. Im, Y., Han, S. H. & Lee, J. Y. Deep blue thermally activated delayed fluorescent emitters using CN-modified indolocarbazole as an acceptor and carbazole-derived donors. *J. Mater. Chem. C* **6**, 5012–5017 (2018).
30. Song, D. et al*.* Asymmetric thermally activated delayed fluorescence (TADF) emitters with 5,9-dioxa-13*b*-boranaphtho[3,2,1-*de*]anthracene (OBA) as acceptor and highly efficient blue-emitting OLEDs. *J. Mater. Chem. C* **7**, 11953–11963 (2019).
31. Stachelek, P. *et al.* Molecular design strategies for color tuning of blue TADF emitters. *ACS Appl. Mater. Interfaces* **11**, 27125–27133 (2019).
